# Supplementary material for: Cytotoxic T cells are able to efficiently eliminate cancer cells by additive cytotoxicity
Source: Nat Commun. 2021 Sep 1;12:5217. doi: 10.1038/s41467-021-25282-3 (PMC8410835; doi:10.1038/s41467-021-25282-3)
Supplement: Supplementary file 1 — Supplementary Information [file 41467_2021_25282_MOESM1_ESM.pdf]

Supplementary Information

Cytotoxic T cells are able to efficiently eliminate cancer cells by additive cytotoxicity

Bettina Weigelin*, Annemieke Th. den Boer, Esther Wagena, Kelly Broen, Harry Dolstra, Rob J de Boer, Carl G. Figdor, Johannes Textor, and Peter Friedl*

*Correspondence to:

bettina.weigelin@med.uni-tuebingen.de
or peter.friedl@radboudumc.nl

Supplementary Figure 1

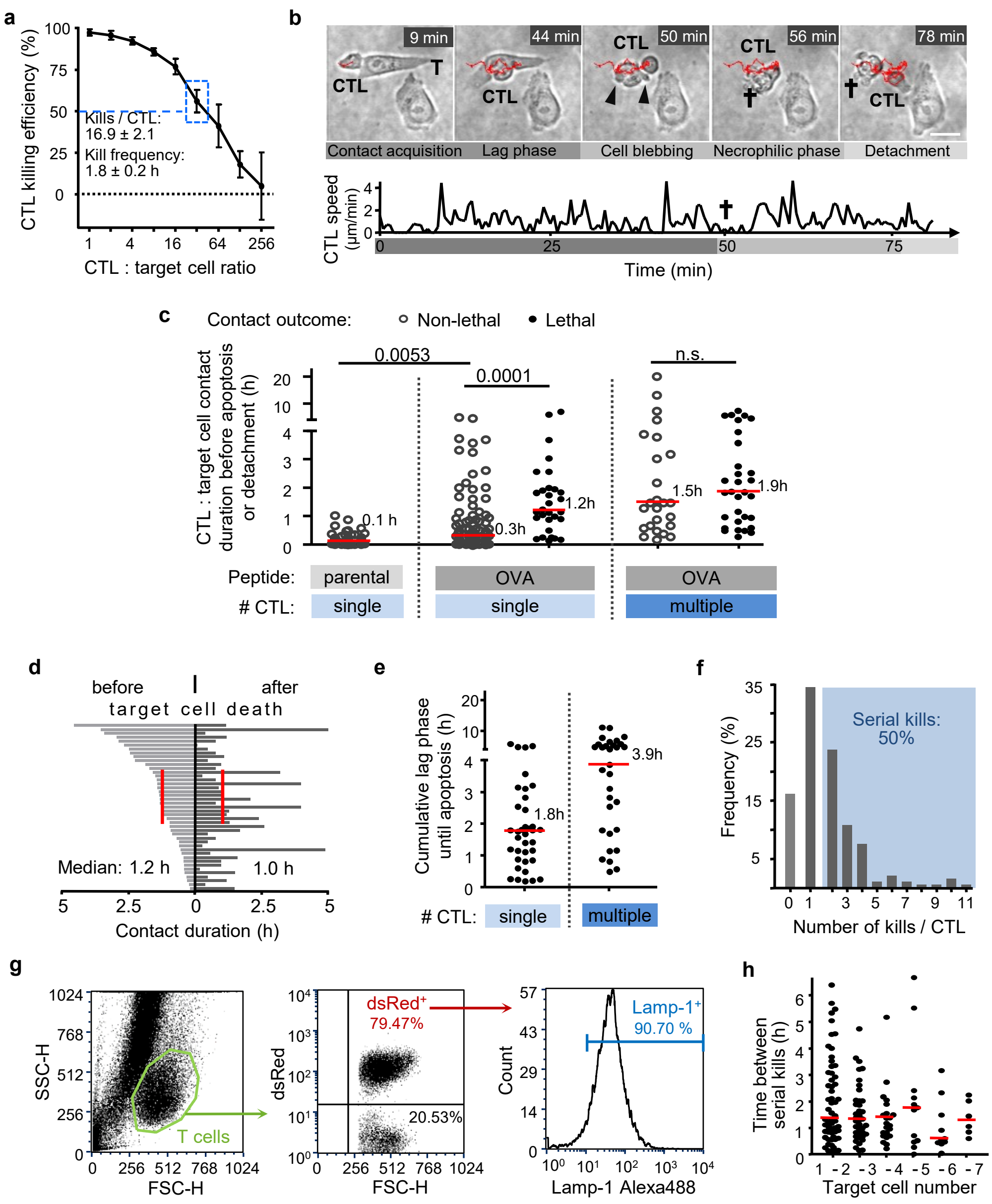

Supplementary Figure 1. CTL contact phases, contact types and duration in the MEC-1/OVA model. a, Killing of MEC-1/OVA targets by OT1 CTL at different ET ratios, determined by flow cytometry after 24 h of co-culture. Data show means ± SD from N = 3 independent experiments. **b,** Image sequence of the different phases of CTL – MEC-1/OVA contact dynamics and apoptosis induction. Scale bar, 10 µm. **c,** Cumulative contact duration between CTL and target cell and contact outcomes. Data from 158 CTL contacts with N = 51 MEC-1 and N = 56 MEC-1/OVA target cells. **d,** Duration of CTL-target cell contacts before and after apoptosis. Data from 20 apoptosis events. **e,** Cumulative lag phase until apoptosis of target cells counted as the time period from the first CTL contact until target cell blebbing in apoptosis events associated with single or multiple CTL interactions. Data from 68 apoptosis events. **f,** Frequency of serial kills. Data from 123 CTL kills. **g,** Gating strategy for Lamp-1 detection on OT1 CTL in coculture assays. T cells were first identified based on their more uniform size distribution compared to tumor cells and subsequently gated on their dsRed^{+} fluorescence. **h,** Time between serial killing events mediated by the same CTL. Data from 155 kills by 39 CTL. *** $p < 0.001$; n.s., not significant (two-tailed Mann-Whitney test). Red bars, median. Data in (**d – f, h**) were pooled from 9 independent experiments. Source data are provided as a Source Data file.

Supplementary Figure 2

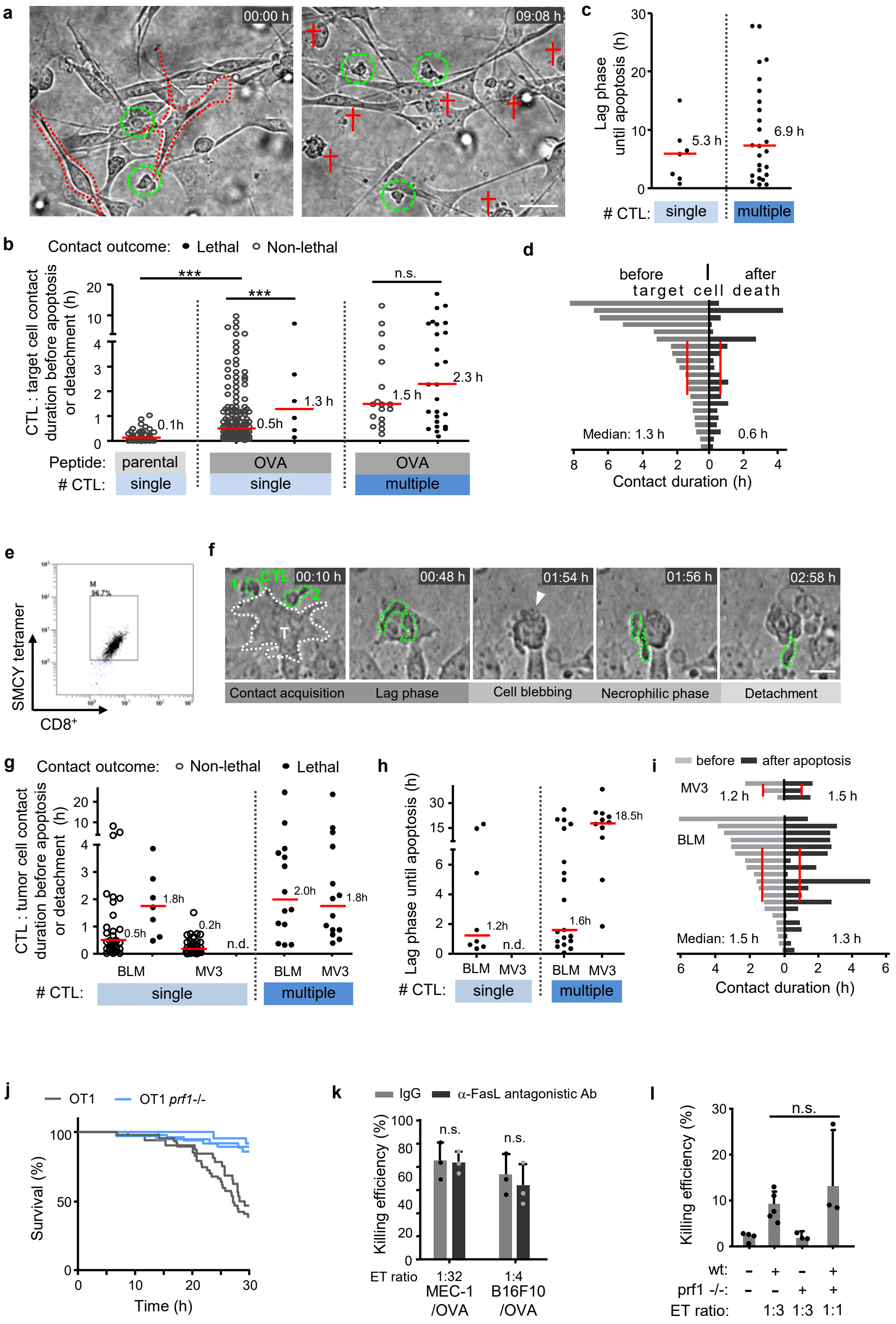

Supplementary Figure 2

Supplementary Figure 2. CTL effector function against mouse B16F10 and human MV3 and BLM melanoma cells. a, Overview image of sub-confluent monolayer of B16F10/OVA with CTL in the 3D collagen matrix interface assay at consecutive time points. Green circles, CTL; red cross, apoptotic target cell. Scale bar, 50 µm. Images show representative example of a time-lapse sequence from the dataset analyzed in b – d. **b,** Duration of contacts between individual and multiple CTL with B16F10/OVA target cells from initiation until apoptosis induction. **c,** Lag phase until apoptosis induced by contacts from single or multiple, sequentially engaged CTL. Data from N = 32 apoptosis events. **d,** Duration of contact phases between individual CTL with target cell before and after target cell blebbing, obtained by manual tracking. Red lines, median. Data in (a-d) show N = 232 contacts pooled from 5 independent experiments; *, $p < 0.01$, ***, $p < 0.0001$, n.s., not significant (two-tailed Mann-Whitney test). **e,** SMCY.A2 tetramer / CD8 double staining confirming CTL specificity and purity of > 95%. **f,** Time-lapse sequence of two simultaneously engaging CTL with human BLM melanoma target cell followed by target-cell apoptosis. Arrowhead, blebbing target cell during death. Scale bar, 20 µm. **g,** SMCY.A2 CTL contact durations with BLM and MV3 melanoma targets and contact outcome. n.d., not detected. **h,** Lag phase until apoptosis induced by single or multiple CTL. **i,** CTL contact duration with the same target cell before and after target cell blebbing. Red bars, median. Data in (g-i) show 50 (MV3) and 53 (BLM) contacts pooled from 3 (MV3) and 4 (BLM) independent experiments. **j,** CTL-mediated killing efficiency of B16F10/OVA cells by wild-type (wt) and perforin-deficient (prf1 -/-) OT1 CTL at 1:2 ET ratio in 3D culture. Data acquisition and analyses as in Fig. 1d. Data show duplicates of one experiment. **k,** CTL killing efficiency upon blocking FasL by α-FasL Ab (MFL4) (10µg/ml). Data represent the means ± SD (n=3). n.s., non-significant (two-tailed Mann-Whitney test). **l**, B16F10/OVA Histone-2B-mCherry / NLS-GFP cocultures with wild type (wt) and mixed, wt and prf1 -/- OT1 CTL were monitored for 40 h by confocal time-lapse microscopy. Readout of CTL-mediated apoptosis induction was achieved by manual analysis of nuclear condensation and fragmentation. Data represent the means ± SD of 171 apoptosis events analyzed in N = 3 independent experiments. ns, non-significant, p-value, two-tailed Mann-Whitney test. Source data are provided as a Source Data file.

Supplementary Figure 3

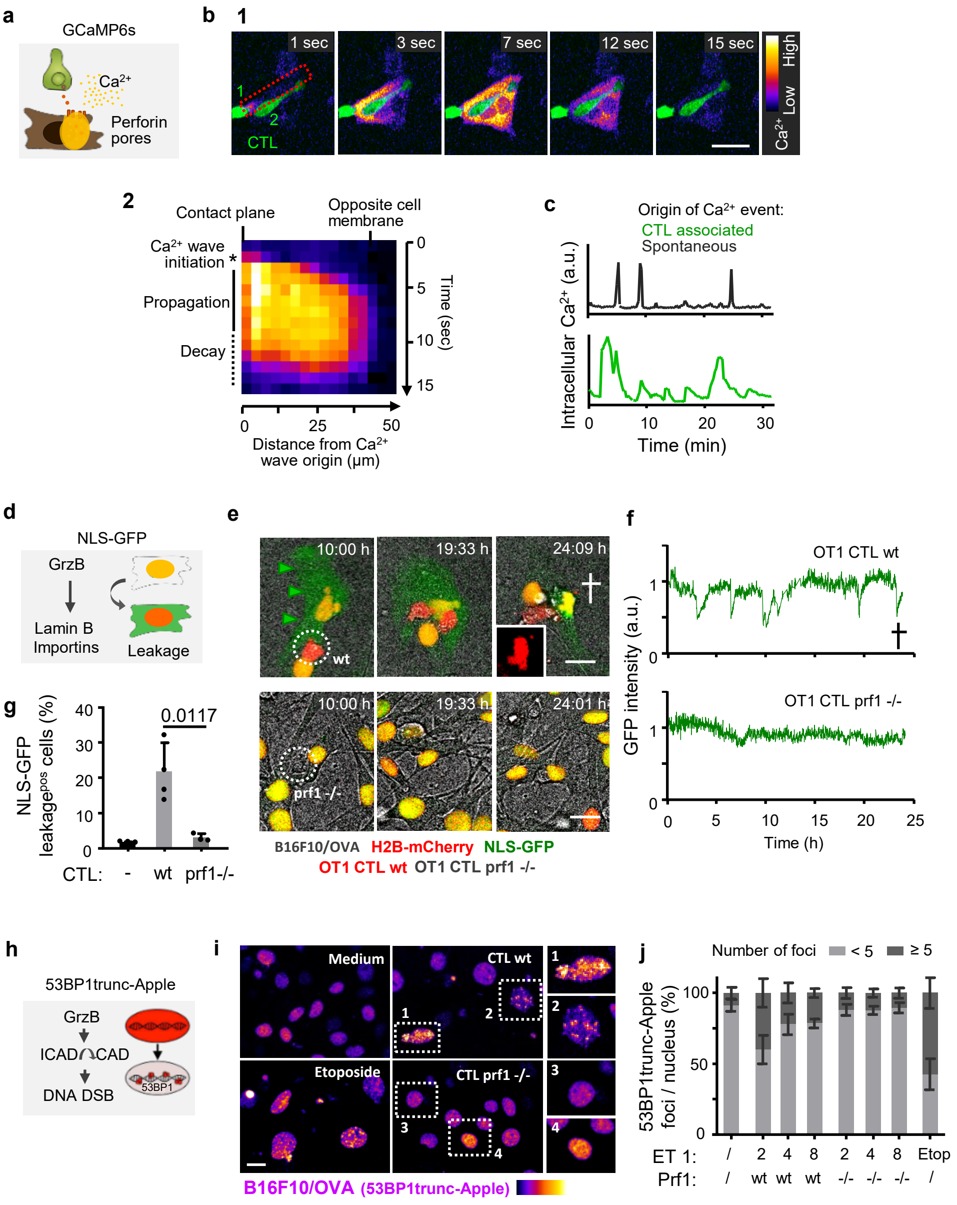

Supplementary Figure 3

Supplementary Figure 3. CTL-mediated sublethal damage kinetics. a, Principle of reporter assay (cartoon, left) and time-lapse sequence of OT1 CTL (green) associated Ca^{2+} event (Fire LUT) in MEC-1/OVA target cell. Frame rate: 1 sec. **b,** Ca^{2+} influx overview sequence (**1**) and detail on the origin at the CTL-target cell contact plane and propagating through the target cell cytoplasm. **c,** Representative example of amplitude and duration of CTL-associated calcium events compared with spontaneous intracellular Ca^{2+} fluctuations in the absence of contacting CTL. **d,** Principle of the NLS-GFP reporter for monitoring sublethal damage. Abbreviations: GrzB, Granzyme B; Yellow nucleus resulting from overlapping emission of NLS-GFP and H2B-mCherry in the nucleus; red nucleus, persisting H2B signal after leakage of NLS-GFP into the cytoplasm. **e,** Representative image sequence of wt and prf1-/- OT1 CTL interacting with NLS-GFP expressing target cells. Dotted circle, CTL; white cross, target cell apoptosis; inset, condensed, apoptotic nucleus (H2B-mCherry). **f,** NLS-GFP signal intensity measured in the nucleus over time and outcome. The GFP signal was divided by the H2B-mCherry signal to normalize for focus drifts which affect signal intensity. Cross, nuclear fragmentation. **g,** Percentage of NLS-GFP leakage events in cultures without or with wt or prf1-/- OT1 CTL. Data show the means ± SD from N = 3 independent experiments. p value, two-tailed Mann-Whitney test. **h,** Principle of the 53BP1trunc-Apple DNA damage reporter. Abbreviations: GrzB, Granzyme B; ICAD/CAD, Inhibitor of Caspase-activated DNase; DNA DSB, DNA double-strand breaks; **i,** Representative micrographs of 53BP1trunc-Apple expressing B16F01/OVA after incubation for 48 h with medium, Etoposide (25 µg/ml), or in coculture with wt or prf1-/- OT1 CTL at ET ratio of 1:4. Zoomed images, nuclei with or without 53BP1trunc-Apple foci. **j,** Percentage of 53BP1trunc-Apple expressing B16F10/OVA cells showing <5 or ≥5 53BP1trunc-Apple foci per nucleus after 48 h culture under different conditions. Foci per nucleus were counted using a custom ImageJ/FIJI script to segment nuclei based on Hoechst counterstaining as the number of maxima per nucleus (Find Maxima plugin). Data show the means ± SD (duplicates from N = 3 independent experiments). Scale bars, 20 µm. Source data are provided as a Source Data file.

Supplementary Figure 4

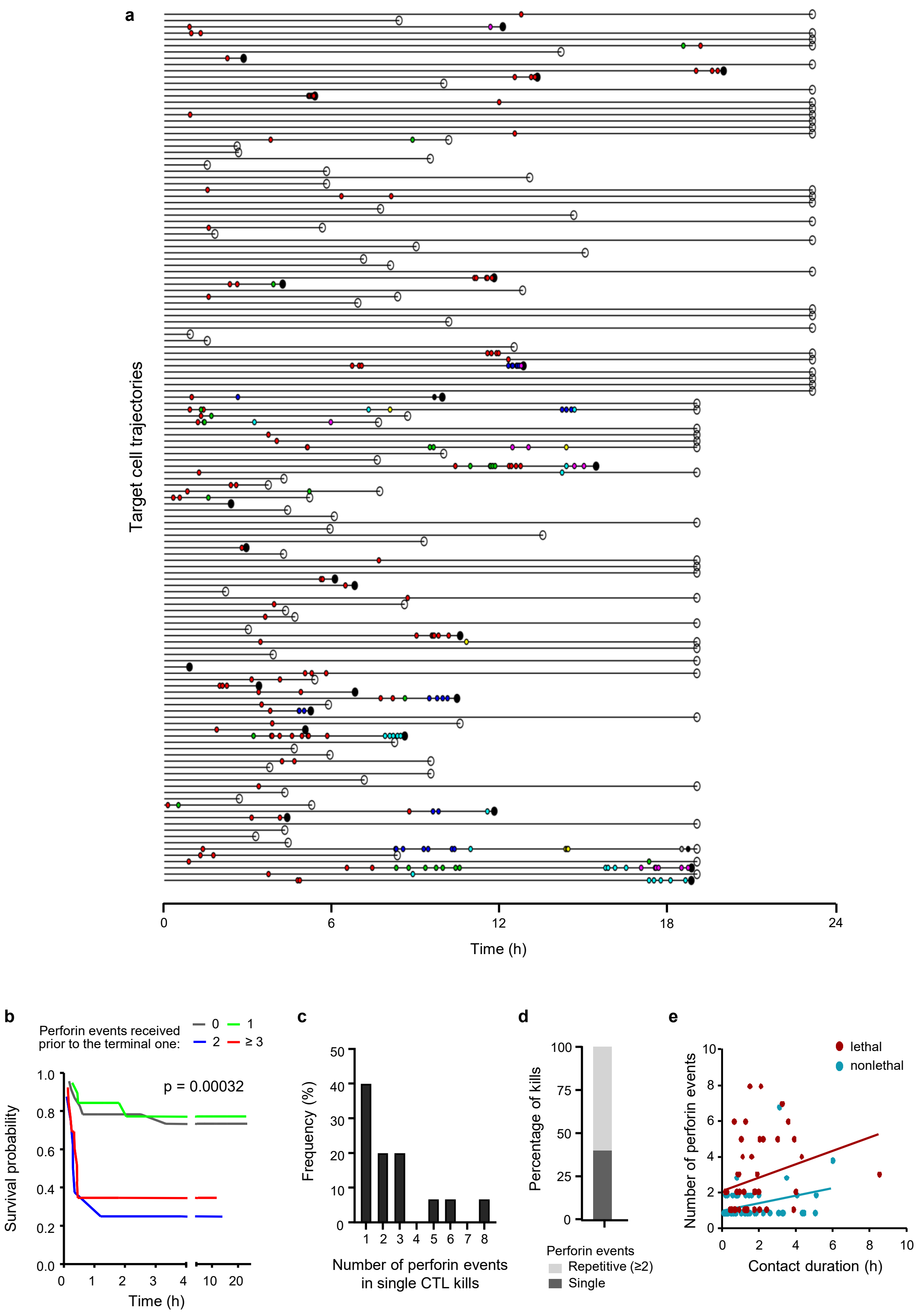

Supplementary Figure 4

Supplementary Figure 4. Statistical analysis. a, Time-points of perforin events (dots color-coded for CTL causing the event) in individual B16F10/OVA cells contacted by OT-1 CTL. Outcome of the interaction is indicated as lethal (filled black dot) or non-lethal (open circle) at the end of each trajectory. **b**, Survival probability of B16F10/OVA cells having received increasing numbers of perforin events prior to the terminal event after filtering redundant perforin events. p-values, two-sided log rank test comparing all groups. Data from 124 perforin events related to 63 B16F10/OVA apoptosis events, pooled from 5 independent experiments. **c,** Frequency distribution of the number of perforin events and **(d)** percentage of single vs. repetitive perforin events in single CTL contacts preceding target cell death. Data represent 15 single CTL interactions from the dataset shown in Fig. S4a. **e,** Correlation of the contact duration and the number of perforin events in lethal or non-lethal interactions quantified from all, single or multiple CTL interactions. Lines, linear regression. Lethal, Spearman r 0.4151, $p < 0.0046$; nonlethal, Spearman r 0.3002, $p < 0.0151$. 110 contacts between B16F10/OVA and OT1 CTL from 3 independent experiments. Source data are provided as a Source Data file.

Supplementary Figure 5

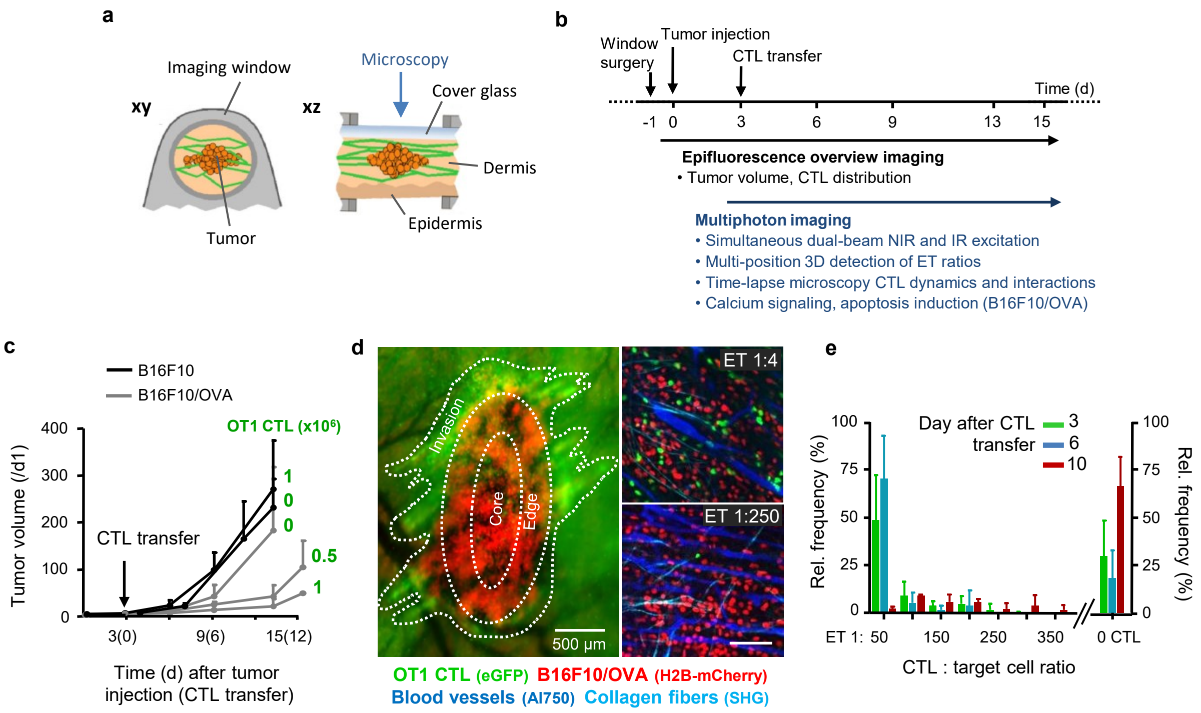

Supplementary Figure 5. Intravital microscopy. a, Dorsal skin-fold chamber setup and tumor model. **b,** Setup and timeline, including tumor cell injection, CTL i.v. transfer, intravital monitoring and parameter extraction. **c,** Time-dependent impact of adoptive OT1 CTL transfer on B16F10 tumor volume based on measurements obtained from epifluorescence overviews. Error bars, means ± SD from N = 4 independent tumors. **d,** Tumor morphology, delineation of subregions and distribution of CTL monitored by epifluorescence detection through the skin window (left image) and multi-photon microscopy images (right images) recorded in different tumor subregions. Red, tumor nuclei (H2B/mCherry); green, OT1 CTL (eGFP); cyan, collagen fibers (SHG); blue, blood vessels (70kDa-dextran/Alexa750). Representative example of CTL tumor infiltration from the dataset analyzed in c and e. **e,** Sub-regional quantification of ET ratios over time. Error bars, means ± SD (d, e, N = 8 independent tumors). Source data are provided as a Source Data file.

Supplementary Figure 6

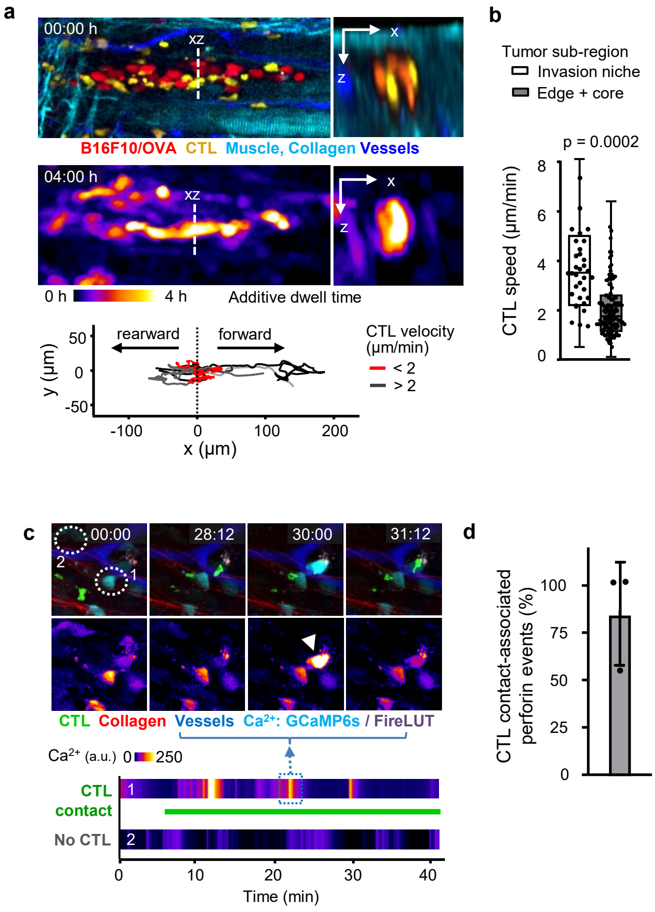

Supplementary Figure 6. Monitoring dynamic cellular events by intravital time-lapse microscopy. a, Distribution (top), dwell-time (middle) and migration tracks (bottom) after 4 h time-lapse recording (top) of CTL (yellow) between tumor cells (red) along channel-like invasion niche confined by collagen bundles and myofibers (cyan, SHG) and blood vessels (blue). Data from one representative time-lapse recording. **b,** CTL migration speed in relation to tumor subregions. Data shows N = 34 (invasion) and 120 (tumor edge) CTL tracks from 3 independent experiments. p value, two-tailed Mann-Whitney test. **c,** Sequential intracellular perforin-mediated Ca^{2+} events (GCaMP6s) in B16F10/OVA cell during CTL engagement. Time stamp, min:sec. Green, OT1 CTL; Cyan, GCaMP6s in B16F10/OVA; Lower panel: Fire LUT, Ca^{2+} intensity (GCaMP6s) of either contact indicated by number. **d**, Percentage of Ca^{2+} events associated with a CTL contact. Error bars, mean ± SD, data from 47 Ca^{2+} events from N = 4 independent mice. Source data are provided as a Source Data file.

Supplementary Figure 7

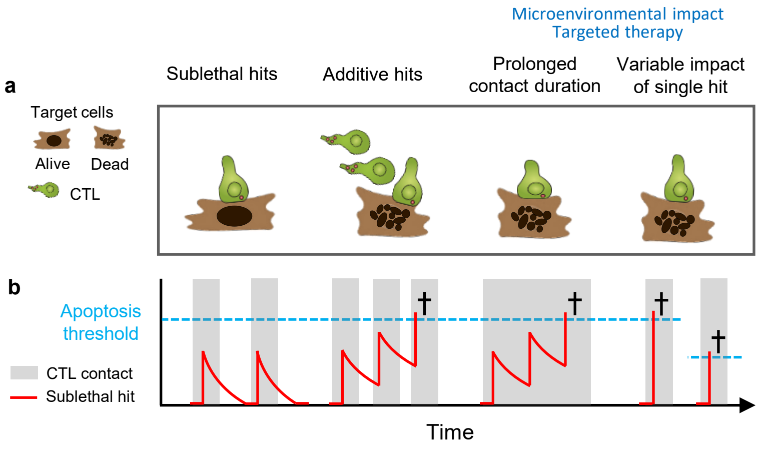

Supplementary Figure 7. The concept of additive cytotoxicity. a, CTL-target cell contact types and outcome. **b,** Predicted accumulation of cytotoxic but sublethal hits and recovery over time. Black cross, target cell apoptosis.
