# Supplementary material for: Cytotoxic T cells are able to efficiently eliminate cancer cells by additive cytotoxicity
Source: Nat Commun. 2021 Sep 1;12:5217. doi: 10.1038/s41467-021-25282-3 (PMC8410835; doi:10.1038/s41467-021-25282-3)
Supplement: Supplementary file 3 — Description of Additional Supplementary Files [file 41467_2021_25282_MOESM3_ESM.pdf]

Description of Additional Supplementary Files

File Name: Supplementary Movie 1
Description: Part 1: B16F10/OVA mouse melanoma target cells cocultured with OT1 CTL. Time, hours:min. Field size 340 x 280 µm. Part 2: BLM human melanoma target cells cocultured with SMCY.A2 CTL. Time, hours:min. Field size 280 x 270 µm.

File Name: Supplementary Movie 2
Description: B16F10/OVA target cell expressing the Ca^{2+} sensor GCaMP6s (Fire LUT) contacted by an OT1 CTL (green). A single short-lived Ca^{2+} event followed by target cell survival (part 1). Repetitive Ca^{2+} events preceding target cell apoptosis (part 2). Time, hours:min:sec. Field size 60 x 55 µm (part 1), 60 x 45 µm (part 2).

File Name: Supplementary Movie 3
Description: Confocal time sequence of B16F10/OVA target cell expressing the NLS-GFP reporter (green) and H2B-mCherry (red) during contact by OT1 CTL (unlabeled, brightfield channel), causing sequential NLS-GFP leakage events with or without apoptosis induction. Circles indicate leakage events. Time, hours:min. Field size 180 x 180 µm.

File Name: Supplementary Movie 4
Description: Confocal time sequence of B16F10/OVA target cell expressing the 53BP1trunc-Apple reporter (Fire LUT) attacked by OT1 CTL (unlabeled), causing 53BP1trunc-Apple focalization followed by resolution (part 1) or apoptosis induction (part 2). Arrowheads indicate DNA repair foci. Time, hours:min. Field size 60 x 60 µm (part 1), 80 x 80 µm (part 2).

File Name: Supplementary Movie 5
Description: Serial engagements of multiple OT1 CTL (green) with B16F10/OVA target cell expressing the Ca^{2+} sensor GCaMP6s (Fire LUT) followed by target cell apoptosis. Time, hours:min:sec. Field size 140 x 85 µm.

File Name: Supplementary Movie 6
Description: Serial engagements of multiple OT1 CTL (dsRed2, yellow) with invading B16F10/OVA target cells (H2B-mCherry nuclei, red) followed by apoptosis of several target cells after multiple CTL contacts. Perfused blood vessels (Al750, blue), Collagen fiber (SHG, cyan). Arrow heads indicate nuclear condensation as first sign of apoptosis. hours:min. Field size 220 x 170 µm.

File Name: Supplementary Movie 7
Description: B16F10/OVA cells expressing the Ca^{2+} sensor GCaMP6s (Cyan/ Fire LUT) in the tumor rim, infiltrated by OT1 CTL (dsRed2, green). CTL conjugation cause sublethal hits in few tumor cells. Perfused blood vessels (Al750, blue), Collagen fiber (SHG, red). Circles indicate Ca^{2+} events. hours:min:sec. Field size 330 x 330 µm.

File Name: Supplementary Movie 8
Description: Tissue invading B16F10/OVA cells expressing the Ca^{2+} sensor GCaMP6s (Cyan/ Fire LUT) serially contacted by OT1 CTL (dsRed2, green). Dynamic CTL conjugations cause repetitive Ca^{2+} events in a high percentage of tumor cells. Perfused blood vessels (Al750, blue), Collagen fiber (SHG, red). Circles indicate Ca^{2+} events. hours:min:sec. Field size 160 x 120 µm.
