# Supplementary material for: Cytotoxic T cells are able to efficiently eliminate cancer cells by additive cytotoxicity
Source: Nat Commun. 2021 Sep 1;12:5217. doi: 10.1038/s41467-021-25282-3 (PMC8410835; doi:10.1038/s41467-021-25282-3)
Supplement: Supplementary file 12 — Reporting Summary [file 41467_2021_25282_MOESM12_ESM.pdf]

nature research

Corresponding author(s): Peter Friedl
Bettina Weigelin

Last updated by author(s): Jun 12, 2021

Reporting Summary

Nature Research wishes to improve the reproducibility of the work that we publish. This form provides structure for consistency and transparency in reporting. For further information on Nature Research policies, see Authors & Referees and the Editorial Policy Checklist .

Statistics

For all statistical analyses, confirm that the following items are present in the figure legend, table legend, main text, or Methods section.

n/a	Confirmed	
☐	☒	The exact sample size (*n*) for each experimental group/condition, given as a discrete number and unit of measurement
☐	☒	A statement on whether measurements were taken from distinct samples or whether the same sample was measured repeatedly
☐	☒	The statistical test(s) used AND whether they are one- or two-sided<br>*Only common tests should be described solely by name; describe more complex techniques in the Methods section.*
☒	☐	A description of all covariates tested
☐	☒	A description of any assumptions or corrections, such as tests of normality and adjustment for multiple comparisons
☐	☒	A full description of the statistical parameters including central tendency (e.g. means) or other basic estimates (e.g. regression coefficient) AND variation (e.g. standard deviation) or associated estimates of uncertainty (e.g. confidence intervals)
☐	☒	For null hypothesis testing, the test statistic (e.g. *F*, *t*, *r*) with confidence intervals, effect sizes, degrees of freedom and *P* value noted<br>*Give P values as exact values whenever suitable.*
☒	☐	For Bayesian analysis, information on the choice of priors and Markov chain Monte Carlo settings
☒	☐	For hierarchical and complex designs, identification of the appropriate level for tests and full reporting of outcomes
☒	☐	Estimates of effect sizes (e.g. Cohen's *d*, Pearson's *r*), indicating how they were calculated

Our web collection on statistics for biologists contains articles on many of the points above.

Software and code

Policy information about availability of computer code

Data collection	No software was used for data collection.
Data analysis	FCS express (version 3 Research Edition; De Novo Software, Los Angeles, CA) and FlowJo (version 9.7.6; FlowJo LLC) were used for the analysis of flow cytometry datasets.<br><br>Bright-field, confocal and two-photon images were processed using FIJI/ImageJ V1.52p (Schindelin et. al. 2012; U.S. National Institutes of Health, Bethesda).<br><br>Prism version 8 for Windows, GraphPad Software (La Jolla California USA, www.graphpad.com) was used for statistical analysis.<br><br>The analysis of the Ca2+ time-to-event data was performed using the R platform for statistical computing, with some preprocessing performed in Python. The R and Python code used for the statistical analysis and simulations of the Ca2+ data was deposited and is publicly available at https://github.com/jtextor/2020-ctl-killing.<br><br>References:<br>Schindelin, J.; Arganda-Carreras, I. & Frise, E. et al. (2012), "Fiji: an open-source platform for biological-image analysis", Nature methods 9 (7): 676-682, PMID 22743772, doi:10.1038/nmeth.2019

For manuscripts utilizing custom algorithms or software that are central to the research but not yet described in published literature, software must be made available to editors/reviewers. We strongly encourage code deposition in a community repository (e.g. GitHub). See the Nature Research guidelines for submitting code & software for further information.

Data

Policy information about availability of data

All manuscripts must include a data availability statement. This statement should provide the following information, where applicable:

- Accession codes, unique identifiers, or web links for publicly available datasets
- A list of figures that have associated raw data
- A description of any restrictions on data availability

The authors declare that data supporting the findings of this study are available within the paper (and its supplementary information files). Primary image data and results will be made available upon reasonable request. Data underlying statistical analyses of sublethal hits are available via github (https://github.com/jtextor/2020-ctl-killing), DOI: http://doi.org/10.5281/zenodo.4922677

Field-specific reporting

Please select the one below that is the best fit for your research. If you are not sure, read the appropriate sections before making your selection.

☒ Life sciences ☐ Behavioural & social sciences ☐ Ecological, evolutionary & environmental sciences

For a reference copy of the document with all sections, see nature.com/documents/nr-reporting-summary-flat.pdf

Life sciences study design

All studies must disclose on these points even when the disclosure is negative.

Sample size	3-10 mice per in vivo condition were analyzed, depending on the effect size of the measured parameter. Group-size calculation was performed per experiment with a power analysis for continuous variables with the program G-Power for in vivo mouse experiments; calculations were made on basis of expected differences and variances from previous in vitro experiments. The exact mouse number per experiment is specified in the figure legends.<br>No sample-size calculation was performed for in vitro experiments. Further details about the sample size are stated in the Figure legends.
Data exclusions	No data were excluded, unless the following exclusion criteria were met:<br>a) the image focus orstable image positioning were lost in long-term time-lapse recordings in vitro<br>b) individual cells did not express the reporter<br>c) there was an inflammation in the imaging window in vivo
Replication	Unless stated otherwise, all experiments were reproduced by at least 3 independent experiments. Further details about the replication of data are stated in the Figure legends.
Randomization	Sample groups tested in vitro were randomly assigned. Mice were randomized for in vivo experiments.
Blinding	Image quantification was performed in a non-blinded, non-anomymized fashion. Blinding was not required because all cells within regions of interest were analyzed in a consistent manner between samples.

Behavioural & social sciences study design

All studies must disclose on these points even when the disclosure is negative.

Study description	*Briefly describe the study type including whether data are quantitative, qualitative, or mixed-methods (e.g. qualitative cross-sectional, quantitative experimental, mixed-methods case study).*
Research sample	*State the research sample (e.g. Harvard university undergraduates, villagers in rural India) and provide relevant demographic information (e.g. age, sex) and indicate whether the sample is representative. Provide a rationale for the study sample chosen. For studies involving existing datasets, please describe the dataset and source.*
Sampling strategy	*Describe the sampling procedure (e.g. random, snowball, stratified, convenience). Describe the statistical methods that were used to predetermine sample size OR if no sample-size calculation was performed, describe how sample sizes were chosen and provide a rationale for why these sample sizes are sufficient. For qualitative data, please indicate whether data saturation was considered, and what criteria were used to decide that no further sampling was needed.*
Data collection	*Provide details about the data collection procedure, including the instruments or devices used to record the data (e.g. pen and paper, computer, eye tracker, video or audio equipment) whether anyone was present besides the participant(s) and the researcher, and whether the researcher was blind to experimental condition and/or the study hypothesis during data collection.*
Timing	*Indicate the start and stop dates of data collection. If there is a gap between collection periods, state the dates for each sample cohort.*

Data exclusions	*If no data were excluded from the analyses, state so OR if data were excluded, provide the exact number of exclusions and the rationale behind them, indicating whether exclusion criteria were pre-established.*
Non-participation	*State how many participants dropped out/declined participation and the reason(s) given OR provide response rate OR state that no participants dropped out/declined participation.*
Randomization	*If participants were not allocated into experimental groups, state so OR describe how participants were allocated to groups, and if allocation was not random, describe how covariates were controlled.*

Ecological, evolutionary & environmental sciences study design

All studies must disclose on these points even when the disclosure is negative.

Study description	*Briefly describe the study. For quantitative data include treatment factors and interactions, design structure (e.g. factorial, nested, hierarchical), nature and number of experimental units and replicates.*
Research sample	*Describe the research sample (e.g. a group of tagged Passer domesticus, all Stenocereus thurberi within Organ Pipe Cactus National Monument), and provide a rationale for the sample choice. When relevant, describe the organism taxa, source, sex, age range and any manipulations. State what population the sample is meant to represent when applicable. For studies involving existing datasets, describe the data and its source.*
Sampling strategy	*Note the sampling procedure. Describe the statistical methods that were used to predetermine sample size OR if no sample-size calculation was performed, describe how sample sizes were chosen and provide a rationale for why these sample sizes are sufficient.*
Data collection	*Describe the data collection procedure, including who recorded the data and how.*
Timing and spatial scale	*Indicate the start and stop dates of data collection, noting the frequency and periodicity of sampling and providing a rationale for these choices. If there is a gap between collection periods, state the dates for each sample cohort. Specify the spatial scale from which the data are taken*
Data exclusions	*If no data were excluded from the analyses, state so OR if data were excluded, describe the exclusions and the rationale behind them, indicating whether exclusion criteria were pre-established.*
Reproducibility	*Describe the measures taken to verify the reproducibility of experimental findings. For each experiment, note whether any attempts to repeat the experiment failed OR state that all attempts to repeat the experiment were successful.*
Randomization	*Describe how samples/organisms/participants were allocated into groups. If allocation was not random, describe how covariates were controlled. If this is not relevant to your study, explain why.*
Blinding	*Describe the extent of blinding used during data acquisition and analysis. If blinding was not possible, describe why OR explain why blinding was not relevant to your study.*

Did the study involve field work? ☐ Yes ☐ No

Field work, collection and transport

Field conditions	*Describe the study conditions for field work, providing relevant parameters (e.g. temperature, rainfall).*
Location	*State the location of the sampling or experiment, providing relevant parameters (e.g. latitude and longitude, elevation, water depth).*
Access and import/export	*Describe the efforts you have made to access habitats and to collect and import/export your samples in a responsible manner and in compliance with local, national and international laws, noting any permits that were obtained (give the name of the issuing authority, the date of issue, and any identifying information).*
Disturbance	*Describe any disturbance caused by the study and how it was minimized.*

Reporting for specific materials, systems and methods

We require information from authors about some types of materials, experimental systems and methods used in many studies. Here, indicate whether each material, system or method listed is relevant to your study. If you are not sure if a list item applies to your research, read the appropriate section before selecting a response.

Materials & experimental systems

n/a	Involved in the study
☐	☒ Antibodies
☐	☒ Eukaryotic cell lines
☐	☐ Palaeontology
☐	☒ Animals and other organisms
☐	☐ Human research participants
☐	☐ Clinical data

Methods

n/a	Involved in the study
☐	☐ ChIP-seq
☐	☒ Flow cytometry
☐	☐ MRI-based neuroimaging

Antibodies

Antibodies used	AlexaFluor488-conjugated anti-Lamp-1 rat-IgG (BIOLEGEND, 121608)<br>Donkey anti-rat/Alexa488 (LIFE TECHNOLOGIES, A21208)<br>IFNγ antagonistic antibody (XMG1.2, Affymetrix 16-7311)<br>Vbeta2<br>CD8<br>CD62L<br>CD44
Validation	For background controls, isotype-matched unspecific mouse IgGs were used for monoclonal antibody stainings,

Eukaryotic cell lines

Policy information about cell lines

Cell line source(s)	B16F10 (kind gift from Gosse Adema, Radboud University Nijmegen, The Netherlands)<br>MEC-1 (kind gift from Stefan Schoenberger, La Jolla Institute for Allergy and Immunology, San Diego, CA, USA)<br>MCF-7 (HTB-22, ATTC)<br>MV3, BLM (kind git of G. van Muijen, Nijmegen; Patient-derived melanoma cell lines established at Radboud University Medical Centre, Nijmegen, the Netherlands; van Muijen GN, et al, Int J Cancer, 1991; van Muijen GN, et al, Clin Exp Metastasis, 1991)
Authentication	The identity of all tumor cells was verified by Short Tandem Repeat (STR) DNA profiling (IDEXX BioResearch). No interspecies contamination was detected.
Mycoplasma contamination	Analysis for mycoplasma contamination was routinely performed for the cell lines (MycoAlert™ Mycoplasma Detection Kit, Lonza).
Commonly misidentified lines<br>(See ICLAC register)	MCF-7 is listed as commonly misidentified cell line. We confirmed the correct identity by STR analysis.

Palaeontology

Specimen provenance	*Provide provenance information for specimens and describe permits that were obtained for the work (including the name of the issuing authority, the date of issue, and any identifying information).*
Specimen deposition	*Indicate where the specimens have been deposited to permit free access by other researchers.*
Dating methods	*If new dates are provided, describe how they were obtained (e.g. collection, storage, sample pretreatment and measurement), where they were obtained (i.e. lab name), the calibration program and the protocol for quality assurance OR state that no new dates are provided.*

☐ Tick this box to confirm that the raw and calibrated dates are available in the paper or in Supplementary Information.

Animals and other organisms

Policy information about studies involving animals; ARRIVE guidelines recommended for reporting animal research

Laboratory animals	C57BL/6 J mice (4-6 weeks of age) were purchased at Charles River Laboratories. Transgenic mice expressing eGFP under the human ubiquitin C promoter (Jackson Laboratories, C57BL/6-Tg(UBC-GFP)30Scha/J, stock number: 004353) and transgenic mice expressing dsRed under the chicken beta-actin promoter (Jackson Laboratories, STOCK Tg(CAG-DsRed*MST)1Nagy/J, stock number: 006051) were crossed to OT-1 TCR transgenic mice (Jackson Laboratories, C57BL/6-Tg(TcraTcrb)1100Mjb/J, stock number: 003831). Double transgenic eGFP/OT-1 and dsRed/OT-1 were bred in the Central Animal Laboratory of the Radboud University Nijmegen, The Netherlands.<br>C57BL/6-Prf1tm1Sdz/J (breeding pairs) were purchased at Jackson Laboratories (Stock No: 002407) and crossed to double-transgenic dsRed/OT-1 mice in the Central Animal Laboratory of the Radboud University Nijmegen, The Netherlands. Genotyping

	was performed by PCR following the genotyping protocol recommended by Jackson Laboratory and mice homozygous for mutant perforin-1 were used for experiments at 8 – 10 weeks of age.
Wild animals	n.a.
Field-collected samples	n.a.
Ethics oversight	All experiments were approved by the Ethical Committee on Animal Experiments and performed in the Central Animal Laboratory of the Radboud University, Nijmegen (RU-DEC 2009-174, 2011-298, 2017-0034), in accordance with the Dutch Animal Experimentation Act and the European FELASA protocol (www.felasa.eu/guidelines.php).

Note that full information on the approval of the study protocol must also be provided in the manuscript.

Human research participants

Policy information about studies involving human research participants

Population characteristics	*Describe the covariate-relevant population characteristics of the human research participants (e.g. age, gender, genotypic information, past and current diagnosis and treatment categories). If you filled out the behavioural & social sciences study design questions and have nothing to add here, write "See above."*
Recruitment	*Describe how participants were recruited. Outline any potential self-selection bias or other biases that may be present and how these are likely to impact results.*
Ethics oversight	*Identify the organization(s) that approved the study protocol.*

Note that full information on the approval of the study protocol must also be provided in the manuscript.

Clinical data

Policy information about clinical studies

All manuscripts should comply with the ICMJEguidelines for publication of clinical research and a completedCONSORT checklist must be included with all submissions.

Clinical trial registration	*Provide the trial registration number from ClinicalTrials.gov or an equivalent agency.*
Study protocol	*Note where the full trial protocol can be accessed OR if not available, explain why.*
Data collection	*Describe the settings and locales of data collection, noting the time periods of recruitment and data collection.*
Outcomes	*Describe how you pre-defined primary and secondary outcome measures and how you assessed these measures.*

ChIP-seq

Data deposition

☐ Confirm that both raw and final processed data have been deposited in a public database such as GEO.

☐ Confirm that you have deposited or provided access to graph files (e.g. BED files) for the called peaks.

Data access links<br>*May remain private before publication.*	*For "Initial submission" or "Revised version" documents, provide reviewer access links. For your "Final submission" document, provide a link to the deposited data.*
Files in database submission	*Provide a list of all files available in the database submission.*
Genome browser session<br>(e.g. UCSC)	*Provide a link to an anonymized genome browser session for "Initial submission" and "Revised version" documents only, to enable peer review. Write "no longer applicable" for "Final submission" documents.*

Methodology

Replicates	*Describe the experimental replicates, specifying number, type and replicate agreement.*
Sequencing depth	*Describe the sequencing depth for each experiment, providing the total number of reads, uniquely mapped reads, length of reads and whether they were paired- or single-end.*
Antibodies	*Describe the antibodies used for the ChIP-seq experiments; as applicable, provide supplier name, catalog number, clone name, and lot number.*
Peak calling parameters	*Specify the command line program and parameters used for read mapping and peak calling, including the ChIP, control and index files used.*
Data quality	*Describe the methods used to ensure data quality in full detail, including how many peaks are at FDR 5% and above 5-fold enrichment.*

Software

Describe the software used to collect and analyze the ChIP-seq data. For custom code that has been deposited into a community repository, provide accession details.

Flow Cytometry

Plots

Confirm that:

- [x] The axis labels state the marker and fluorochrome used (e.g. CD4-FITC).
- [x] The axis scales are clearly visible. Include numbers along axes only for bottom left plot of group (a 'group' is an analysis of identical markers).
- [x] All plots are contour plots with outliers or pseudocolor plots.
- [x] A numerical value for number of cells or percentage (with statistics) is provided.

Methodology

Sample preparation	Flow cytometry was used to detect Lamp-1 expression on the T cell surface after incubation with target cells in 3D killing assays. CTL and surviving target cells were harvested by dissolving the collagen with collagenase I (40 U / 96-well; 30 min; SIGMA C0130) and detaching the remaining adherent cells with trypsin/EDTA (5 min). Both cell fractions were combined, washed in PBS, stained with AlexaFluor488-conjugated anti-Lamp-1 rat-IgG (BIOLEGEND, 121608) and detected after signal enhancement by donkey anti-rat/Alexa488 (LIFE TECHNOLOGIES, A21208).
Instrument	BD FACSCalibur, BD Biosciences, Erembodegem, Belgium
Software	FCS Express (Version 3 Research Edition; De Novo Software, Los Angeles, CA)
Cell population abundance	10,000 - 20,000 cells per condition were selected after gating strategy.
Gating strategy	CTL were gated on intact morphology, viability by propidium iodide exclusion and dsRed expression using De Novo FCS Express 3. Percentages of positive events were calculated using the build-in FCS Express function for histogram subtraction.

- [x] Tick this box to confirm that a figure exemplifying the gating strategy is provided in the Supplementary Information.

Magnetic resonance imaging

Experimental design

Design type	*Indicate task or resting state; event-related or block design.*
Design specifications	*Specify the number of blocks, trials or experimental units per session and/or subject, and specify the length of each trial or block (if trials are blocked) and interval between trials.*
Behavioral performance measures	*State number and/or type of variables recorded (e.g. correct button press, response time) and what statistics were used to establish that the subjects were performing the task as expected (e.g. mean, range, and/or standard deviation across subjects).*

Acquisition

Imaging type(s)	*Specify: functional, structural, diffusion, perfusion.*
Field strength	*Specify in Tesla*
Sequence & imaging parameters	*Specify the pulse sequence type (gradient echo, spin echo, etc.), imaging type (EPI, spiral, etc.), field of view, matrix size, slice thickness, orientation and TE/TR/flip angle.*
Area of acquisition	*State whether a whole brain scan was used OR define the area of acquisition, describing how the region was determined.*

Diffusion MRI ☐ Used ☐ Not used

Preprocessing

Preprocessing software	*Provide detail on software version and revision number and on specific parameters (model/functions, brain extraction, segmentation, smoothing kernel size, etc.).*
Normalization	*If data were normalized/standardized, describe the approach(es): specify linear or non-linear and define image types used for transformation OR indicate that data were not normalized and explain rationale for lack of normalization.*
Normalization template	*Describe the template used for normalization/transformation, specifying subject space or group standardized space (e.g. original Talairach, MNI305, ICBM152) OR indicate that the data were not normalized.*

Noise and artifact removal

Describe your procedure(s) for artifact and structured noise removal, specifying motion parameters, tissue signals and physiological signals (heart rate, respiration).

Volume censoring

Define your software and/or method and criteria for volume censoring, and state the extent of such censoring.

Statistical modeling & inference

Model type and settings

Specify type (mass univariate, multivariate, RSA, predictive, etc.) and describe essential details of the model at the first and second levels (e.g. fixed, random or mixed effects; drift or auto-correlation).

Effect(s) tested

Define precise effect in terms of the task or stimulus conditions instead of psychological concepts and indicate whether ANOVA or factorial designs were used.

Specify type of analysis: ☐ Whole brain ☐ ROI-based ☐ Both

Statistic type for inference
(See Eklund et al. 2016)

Specify voxel-wise or cluster-wise and report all relevant parameters for cluster-wise methods.

Correction

Describe the type of correction and how it is obtained for multiple comparisons (e.g. FWE, FDR, permutation or Monte Carlo).

Models & analysis

n/a	Involved in the study
☐	☐ Functional and/or effective connectivity
☐	☐ Graph analysis
☐	☐ Multivariate modeling or predictive analysis

Functional and/or effective connectivity

Report the measures of dependence used and the model details (e.g. Pearson correlation, partial correlation, mutual information).

Graph analysis

Report the dependent variable and connectivity measure, specifying weighted graph or binarized graph, subject- or group-level, and the global and/or node summaries used (e.g. clustering coefficient, efficiency, etc.).

Multivariate modeling and predictive analysis

Specify independent variables, features extraction and dimension reduction, model, training and evaluation metrics.
